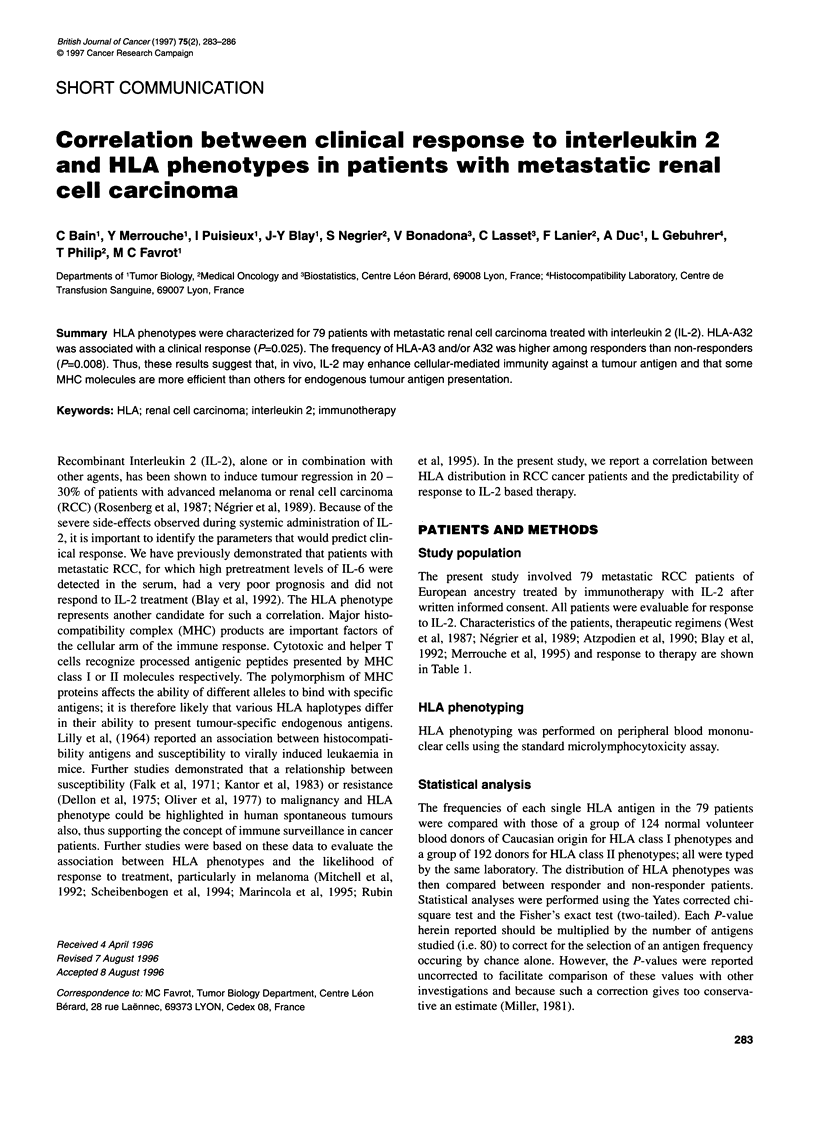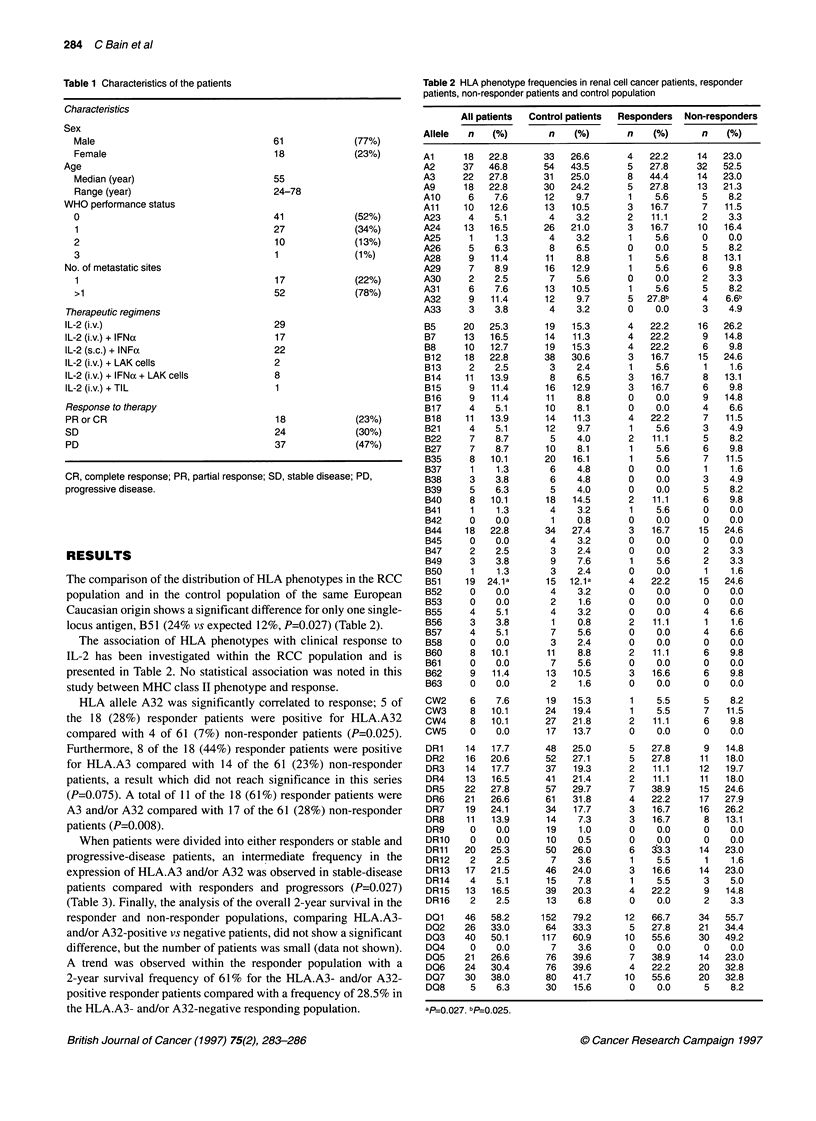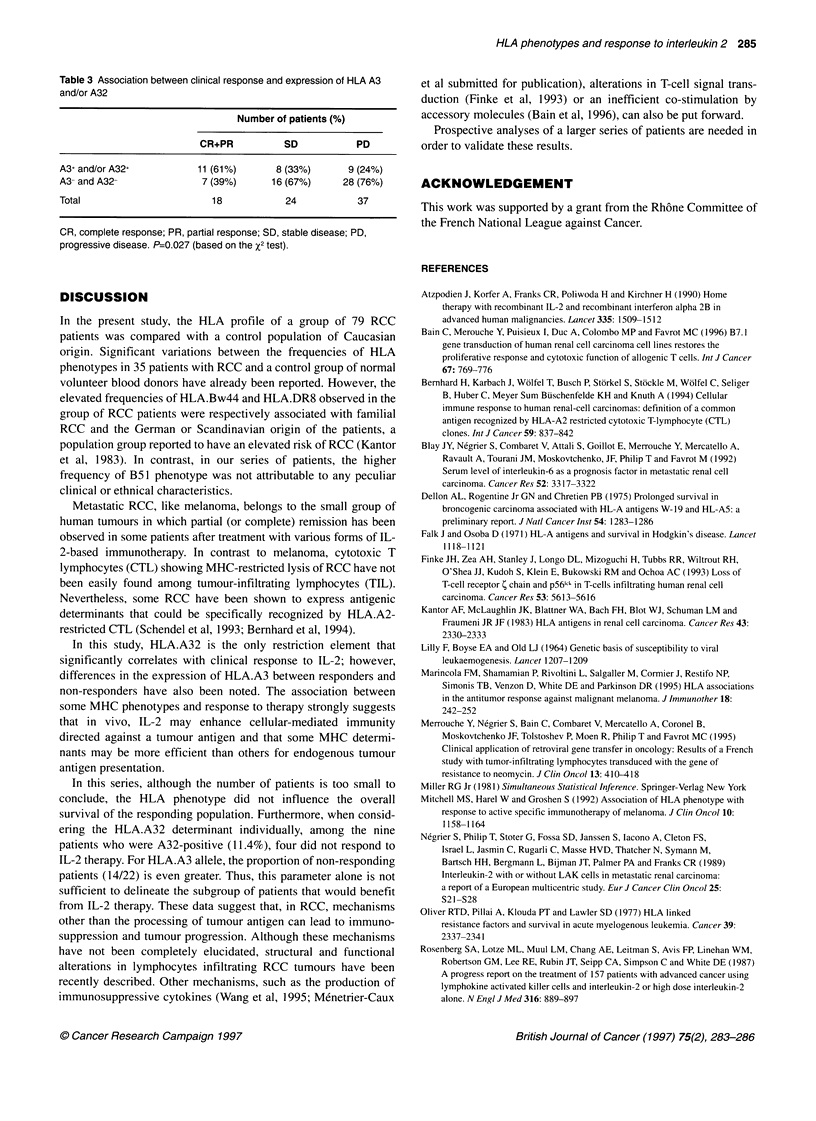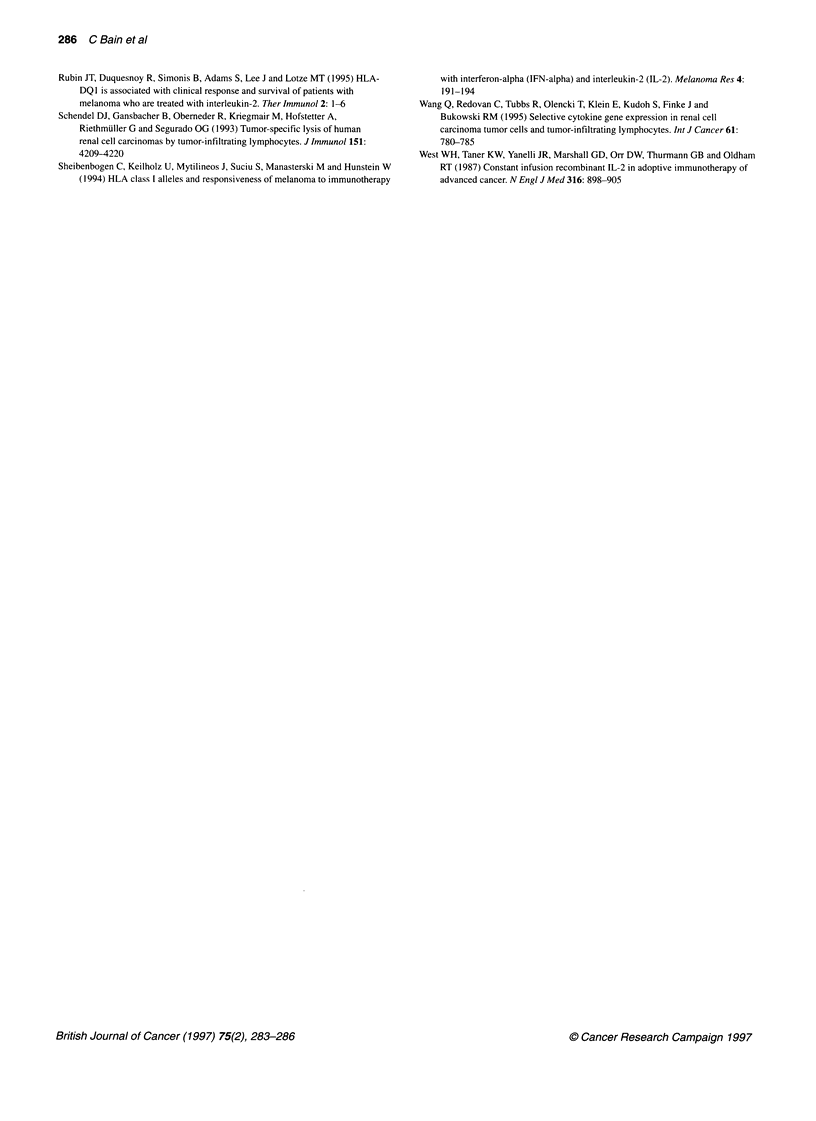# Correlation between clinical response to interleukin 2 and HLA phenotypes in patients with metastatic renal cell carcinoma.

**DOI:** 10.1038/bjc.1997.46

**Published:** 1997

**Authors:** C. Bain, Y. Merrouche, I. Puisieux, J. Y. Blay, S. Negrier, V. Bonadona, C. Lasset, F. Lanier, A. Duc, L. Gebuhrer, T. Philip, M. C. Favrot

**Affiliations:** Department of Tumor Biology, Centre Léon Bérard, Lyon, France.

## Abstract

HLA phenotypes were characterized for 79 patients with metastatic renal cell carcinoma treated with interleukin 2 (IL-2). HLA-A32 was associated with a clinical response (P = 0.025). The frequency of HLA-A3 and/or A32 was higher among responders than non-responders (P = 0.008). Thus, these results suggest that, in vivo, IL-2 may enhance cellular-mediated immunity against a tumour antigen and that some MHC molecules are more efficient than others for endogenous tumour antigen presentation.


British Journal of Cancer (1997) 75(2), 283–286
© 1997 Cancer Research Campaign

SHORT COMMUNICATION

### Correlation between clinical response to interleukin 2 and HLA phenotypes in patients with metastatic renal cell carcinoma

**C Bain[1], Y Merrouche[1], I Puisieux[1], J-Y Blay[1], S Negrier[2], V Bonadona[3], C Lasset[3], F Lanier[2], A Duc[1], L Gebuhrer[4], T Philip[2], M C Favrot[1]**

Departments of [1]Tumor Biology, [2]Medical Oncology and [3]Biostatistics, Centre Léon Bérard, 69008 Lyon, France; [4]Histocompatibility Laboratory, Centre de Transfusion Sanguine, 69007 Lyon, France

**Summary** HLA phenotypes were characterized for 79 patients with metastatic renal cell carcinoma treated with interleukin 2 (IL-2). HLA-A32 was associated with a clinical response (*P*=0.025). The frequency of HLA-A3 and/or A32 was higher among responders than non-responders (*P*=0.008). Thus, these results suggest that, in vivo, IL-2 may enhance cellular-mediated immunity against a tumour antigen and that some MHC molecules are more efficient than others for endogenous tumour antigen presentation.

**Keywords:** HLA; renal cell carcinoma; interleukin 2; immunotherapy

Recombinant Interleukin 2 (IL-2), alone or in combination with other agents, has been shown to induce tumour regression in 20 – 30% of patients with advanced melanoma or renal cell carcinoma (RCC) (Rosenberg et al, 1987; Négrier et al, 1989). Because of the severe side-effects observed during systemic administration of IL-2, it is important to identify the parameters that would predict clinical response. We have previously demonstrated that patients with metastatic RCC, for which high pretreatment levels of IL-6 were detected in the serum, had a very poor prognosis and did not respond to IL-2 treatment (Blay et al, 1992). The HLA phenotype represents another candidate for such a correlation. Major histocompatibility complex (MHC) products are important factors of the cellular arm of the immune response. Cytotoxic and helper T cells recognize processed antigenic peptides presented by MHC class I or II molecules respectively. The polymorphism of MHC proteins affects the ability of different alleles to bind with specific antigens; it is therefore likely that various HLA haplotypes differ in their ability to present tumour-specific endogenous antigens. Lilly et al, (1964) reported an association between histocompatibility antigens and susceptibility to virally induced leukaemia in mice. Further studies demonstrated that a relationship between susceptibility (Falk et al, 1971; Kantor et al, 1983) or resistance (Dellon et al, 1975; Oliver et al, 1977) to malignancy and HLA phenotype could be highlighted in human spontaneous tumours also, thus supporting the concept of immune surveillance in cancer patients. Further studies were based on these data to evaluate the association between HLA phenotypes and the likelihood of response to treatment, particularly in melanoma (Mitchell et al, 1992; Scheibenbogen et al, 1994; Marincola et al, 1995; Rubin et al, 1995). In the present study, we report a correlation between HLA distribution in RCC cancer patients and the predictability of response to IL-2 based therapy.

*Received 4 April 1996*
*Revised 7 August 1996*
*Accepted 8 August 1996*

*Correspondence to:* MC Favrot, Tumor Biology Department, Centre Léon Bérard, 28 rue Laënnec, 69373 LYON, Cedex 08, France

### PATIENTS AND METHODS

### Study population

The present study involved 79 metastatic RCC patients of European ancestry treated by immunotherapy with IL-2 after written informed consent. All patients were evaluable for response to IL-2. Characteristics of the patients, therapeutic regimens (West et al, 1987; Négrier et al, 1989; Atzpodien et al, 1990; Blay et al, 1992; Merrouche et al, 1995) and response to therapy are shown in Table 1.

### HLA phenotyping

HLA phenotyping was performed on peripheral blood mononuclear cells using the standard microlymphocytoxicity assay.

### Statistical analysis

The frequencies of each single HLA antigen in the 79 patients were compared with those of a group of 124 normal volunteer blood donors of Caucasian origin for HLA class I phenotypes and a group of 192 donors for HLA class II phenotypes; all were typed by the same laboratory. The distribution of HLA phenotypes was then compared between responder and non-responder patients. Statistical analyses were performed using the Yates corrected chi-square test and the Fisher's exact test (two-tailed). Each *P*-value herein reported should be multiplied by the number of antigens studied (i.e. 80) to correct for the selection of an antigen frequency occuring by chance alone. However, the *P*-values were reported uncorrected to facilitate comparison of these values with other investigations and because such a correction gives too conservative an estimate (Miller, 1981).

**Table 1** Characteristics of the patients

| *Characteristics* | | |
|---|---|---|
| Sex | | |
| Male | 61 | (77%) |
| Female | 18 | (23%) |
| Age | | |
| Median (year) | 55 | |
| Range (year) | 24–78 | |
| WHO performance status | | |
| 0 | 41 | (52%) |
| 1 | 27 | (34%) |
| 2 | 10 | (13%) |
| 3 | 1 | (1%) |
| No. of metastatic sites | | |
| 1 | 17 | (22%) |
| >1 | 52 | (78%) |
| *Therapeutic regimens* | | |
| IL-2 (i.v.) | 29 | |
| IL-2 (i.v.) + IFNα | 17 | |
| IL-2 (s.c.) + INFα | 22 | |
| IL-2 (i.v.) + LAK cells | 2 | |
| IL-2 (i.v.) + IFNα + LAK cells | 8 | |
| IL-2 (i.v.) + TIL | 1 | |
| *Response to therapy* | | |
| PR or CR | 18 | (23%) |
| SD | 24 | (30%) |
| PD | 37 | (47%) |

CR, complete response; PR, partial response; SD, stable disease; PD, progressive disease.

### RESULTS

The comparison of the distribution of HLA phenotypes in the RCC population and in the control population of the same European Caucasian origin shows a significant difference for only one single-locus antigen, B51 (24% *vs* expected 12%, $P$=0.027) (Table 2).

The association of HLA phenotypes with clinical response to IL-2 has been investigated within the RCC population and is presented in Table 2. No statistical association was noted in this study between MHC class II phenotype and response.

HLA allele A32 was significantly correlated to response; 5 of the 18 (28%) responder patients were positive for HLA.A32 compared with 4 of 61 (7%) non-responder patients ($P$=0.025). Furthermore, 8 of the 18 (44%) responder patients were positive for HLA.A3 compared with 14 of the 61 (23%) non-responder patients, a result which did not reach significance in this series ($P$=0.075). A total of 11 of the 18 (61%) responder patients were A3 and/or A32 compared with 17 of the 61 (28%) non-responder patients ($P$=0.008).

When patients were divided into either responders or stable and progressive-disease patients, an intermediate frequency in the expression of HLA.A3 and/or A32 was observed in stable-disease patients compared with responders and progressors ($P$=0.027) (Table 3). Finally, the analysis of the overall 2-year survival in the responder and non-responder populations, comparing HLA.A3- and/or A32-positive *vs* negative patients, did not show a significant difference, but the number of patients was small (data not shown). A trend was observed within the responder population with a 2-year survival frequency of 61% for the HLA.A3- and/or A32-positive responder patients compared with a frequency of 28.5% in the HLA.A3- and/or A32-negative responding population.

**Table 2** HLA phenotype frequencies in renal cell cancer patients, responder patients, non-responder patients and control population

| | All patients | | Control patients | | Responders | | Non-responders | |
|---|---|---|---|---|---|---|---|---|
| **Allele** | ***n*** | **(%)** | ***n*** | **(%)** | ***n*** | **(%)** | ***n*** | **(%)** |
| A1 | 18 | 22.8 | 33 | 26.6 | 4 | 22.2 | 14 | 23.0 |
| A2 | 37 | 46.8 | 54 | 43.5 | 5 | 27.8 | 32 | 52.5 |
| A3 | 22 | 27.8 | 31 | 25.0 | 8 | 44.4 | 14 | 23.0 |
| A9 | 18 | 22.8 | 30 | 24.2 | 5 | 27.8 | 13 | 21.3 |
| A10 | 6 | 7.6 | 12 | 9.7 | 1 | 5.6 | 5 | 8.2 |
| A11 | 10 | 12.6 | 13 | 10.5 | 3 | 16.7 | 7 | 11.5 |
| A23 | 4 | 5.1 | 4 | 3.2 | 2 | 11.1 | 2 | 3.3 |
| A24 | 13 | 16.5 | 26 | 21.0 | 3 | 16.7 | 10 | 16.4 |
| A25 | 1 | 1.3 | 4 | 3.2 | 1 | 5.6 | 0 | 0.0 |
| A26 | 5 | 6.3 | 8 | 6.5 | 0 | 0.0 | 5 | 8.2 |
| A28 | 9 | 11.4 | 11 | 8.8 | 1 | 5.6 | 8 | 13.1 |
| A29 | 7 | 8.9 | 16 | 12.9 | 1 | 5.6 | 6 | 9.8 |
| A30 | 2 | 2.5 | 7 | 5.6 | 0 | 0.0 | 2 | 3.3 |
| A31 | 6 | 7.6 | 13 | 10.5 | 1 | 5.6 | 5 | 8.2 |
| A32 | 9 | 11.4 | 12 | 9.7 | 5 | 27.8[b] | 4 | 6.6[b] |
| A33 | 3 | 3.8 | 4 | 3.2 | 0 | 0.0 | 3 | 4.9 |
| B5 | 20 | 25.3 | 19 | 15.3 | 4 | 22.2 | 16 | 26.2 |
| B7 | 13 | 16.5 | 14 | 11.3 | 4 | 22.2 | 9 | 14.8 |
| B8 | 10 | 12.7 | 19 | 15.3 | 4 | 22.2 | 6 | 9.8 |
| B12 | 18 | 22.8 | 38 | 30.6 | 3 | 16.7 | 15 | 24.6 |
| B13 | 2 | 2.5 | 3 | 2.4 | 1 | 5.6 | 1 | 1.6 |
| B14 | 11 | 13.9 | 8 | 6.5 | 3 | 16.7 | 8 | 13.1 |
| B15 | 9 | 11.4 | 16 | 12.9 | 3 | 16.7 | 6 | 9.8 |
| B16 | 9 | 11.4 | 11 | 8.8 | 0 | 0.0 | 9 | 14.8 |
| B17 | 4 | 5.1 | 10 | 8.1 | 0 | 0.0 | 4 | 6.6 |
| B18 | 11 | 13.9 | 14 | 11.3 | 4 | 22.2 | 7 | 11.5 |
| B21 | 4 | 5.1 | 12 | 9.7 | 1 | 5.6 | 3 | 4.9 |
| B22 | 7 | 8.7 | 5 | 4.0 | 2 | 11.1 | 5 | 8.2 |
| B27 | 7 | 8.7 | 10 | 8.1 | 1 | 5.6 | 6 | 9.8 |
| B35 | 8 | 10.1 | 20 | 16.1 | 1 | 5.6 | 7 | 11.5 |
| B37 | 1 | 1.3 | 6 | 4.8 | 0 | 0.0 | 1 | 1.6 |
| B38 | 3 | 3.8 | 6 | 4.8 | 0 | 0.0 | 3 | 4.9 |
| B39 | 5 | 6.3 | 5 | 4.0 | 0 | 0.0 | 5 | 8.2 |
| B40 | 8 | 10.1 | 18 | 14.5 | 2 | 11.1 | 6 | 9.8 |
| B41 | 1 | 1.3 | 4 | 3.2 | 1 | 5.6 | 0 | 0.0 |
| B42 | 0 | 0.0 | 1 | 0.8 | 0 | 0.0 | 0 | 0.0 |
| B44 | 18 | 22.8 | 34 | 27.4 | 3 | 16.7 | 15 | 24.6 |
| B45 | 0 | 0.0 | 4 | 3.2 | 0 | 0.0 | 0 | 0.0 |
| B47 | 2 | 2.5 | 3 | 2.4 | 0 | 0.0 | 2 | 3.3 |
| B49 | 3 | 3.8 | 9 | 7.6 | 1 | 5.6 | 2 | 3.3 |
| B50 | 1 | 1.3 | 3 | 2.4 | 0 | 0.0 | 1 | 1.6 |
| B51 | 19 | 24.1[a] | 15 | 12.1[a] | 4 | 22.2 | 15 | 24.6 |
| B52 | 0 | 0.0 | 4 | 3.2 | 0 | 0.0 | 0 | 0.0 |
| B53 | 0 | 0.0 | 2 | 1.6 | 0 | 0.0 | 0 | 0.0 |
| B55 | 4 | 5.1 | 4 | 3.2 | 0 | 0.0 | 4 | 6.6 |
| B56 | 3 | 3.8 | 1 | 0.8 | 2 | 11.1 | 1 | 1.6 |
| B57 | 4 | 5.1 | 7 | 5.6 | 0 | 0.0 | 4 | 6.6 |
| B58 | 0 | 0.0 | 3 | 2.4 | 0 | 0.0 | 0 | 0.0 |
| B60 | 8 | 10.1 | 11 | 8.8 | 2 | 11.1 | 6 | 9.8 |
| B61 | 0 | 0.0 | 7 | 5.6 | 0 | 0.0 | 0 | 0.0 |
| B62 | 9 | 11.4 | 13 | 10.5 | 3 | 16.6 | 6 | 9.8 |
| B63 | 0 | 0.0 | 2 | 1.6 | 0 | 0.0 | 0 | 0.0 |
| CW2 | 6 | 7.6 | 19 | 15.3 | 1 | 5.5 | 5 | 8.2 |
| CW3 | 8 | 10.1 | 24 | 19.4 | 1 | 5.5 | 7 | 11.5 |
| CW4 | 8 | 10.1 | 27 | 21.8 | 2 | 11.1 | 6 | 9.8 |
| CW5 | 0 | 0.0 | 17 | 13.7 | 0 | 0.0 | 0 | 0.0 |
| DR1 | 14 | 17.7 | 48 | 25.0 | 5 | 27.8 | 9 | 14.8 |
| DR2 | 16 | 20.6 | 52 | 27.1 | 5 | 27.8 | 11 | 18.0 |
| DR3 | 14 | 17.7 | 37 | 19.3 | 2 | 11.1 | 12 | 19.7 |
| DR4 | 13 | 16.5 | 41 | 21.4 | 2 | 11.1 | 11 | 18.0 |
| DR5 | 22 | 27.8 | 57 | 29.7 | 7 | 38.9 | 15 | 24.6 |
| DR6 | 21 | 26.6 | 61 | 31.8 | 4 | 22.2 | 17 | 27.9 |
| DR7 | 19 | 24.1 | 34 | 17.7 | 3 | 16.7 | 16 | 26.2 |
| DR8 | 11 | 13.9 | 14 | 7.3 | 3 | 16.7 | 8 | 13.1 |
| DR9 | 0 | 0.0 | 19 | 1.0 | 0 | 0.0 | 0 | 0.0 |
| DR10 | 0 | 0.0 | 10 | 0.5 | 0 | 0.0 | 0 | 0.0 |
| DR11 | 20 | 25.3 | 50 | 26.0 | 6 | 33.3 | 14 | 23.0 |
| DR12 | 2 | 2.5 | 7 | 3.6 | 1 | 5.5 | 1 | 1.6 |
| DR13 | 17 | 21.5 | 46 | 24.0 | 3 | 16.6 | 14 | 23.0 |
| DR14 | 4 | 5.1 | 15 | 7.8 | 1 | 5.5 | 3 | 5.0 |
| DR15 | 13 | 16.5 | 39 | 20.3 | 4 | 22.2 | 9 | 14.8 |
| DR16 | 2 | 2.5 | 13 | 6.8 | 0 | 0.0 | 2 | 3.3 |
| DQ1 | 46 | 58.2 | 152 | 79.2 | 12 | 66.7 | 34 | 55.7 |
| DQ2 | 26 | 33.0 | 64 | 33.3 | 5 | 27.8 | 21 | 34.4 |
| DQ3 | 40 | 50.1 | 117 | 60.9 | 10 | 55.6 | 30 | 49.2 |
| DQ4 | 0 | 0.0 | 7 | 3.6 | 0 | 0.0 | 0 | 0.0 |
| DQ5 | 21 | 26.6 | 76 | 39.6 | 7 | 38.9 | 14 | 23.0 |
| DQ6 | 24 | 30.4 | 76 | 39.6 | 4 | 22.2 | 20 | 32.8 |
| DQ7 | 30 | 38.0 | 80 | 41.7 | 10 | 55.6 | 20 | 32.8 |
| DQ8 | 5 | 6.3 | 30 | 15.6 | 0 | 0.0 | 5 | 8.2 |

[a]$P$=0.027. [b]$P$=0.025.

© Cancer Research Campaign 1997

**Table 3** Association between clinical response and expression of HLA A3 and/or A32

| | Number of patients (%) | | |
|---|---|---|---|
| | CR+PR | SD | PD |
| $A3^+$ and/or $A32^+$ | 11 (61%) | 8 (33%) | 9 (24%) |
| $A3^-$ and $A32^-$ | 7 (39%) | 16 (67%) | 28 (76%) |
| Total | 18 | 24 | 37 |

CR, complete response; PR, partial response; SD, stable disease; PD, progressive disease. $P$=0.027 (based on the $\chi^2$ test).

### DISCUSSION

In the present study, the HLA profile of a group of 79 RCC patients was compared with a control population of Caucasian origin. Significant variations between the frequencies of HLA phenotypes in 35 patients with RCC and a control group of normal volunteer blood donors have already been reported. However, the elevated frequencies of HLA.Bw44 and HLA.DR8 observed in the group of RCC patients were respectively associated with familial RCC and the German or Scandinavian origin of the patients, a population group reported to have an elevated risk of RCC (Kantor et al, 1983). In contrast, in our series of patients, the higher frequency of B51 phenotype was not attributable to any peculiar clinical or ethnical characteristics.

Metastatic RCC, like melanoma, belongs to the small group of human tumours in which partial (or complete) remission has been observed in some patients after treatment with various forms of IL-2-based immunotherapy. In contrast to melanoma, cytotoxic T lymphocytes (CTL) showing MHC-restricted lysis of RCC have not been easily found among tumour-infiltrating lymphocytes (TIL). Nevertheless, some RCC have been shown to express antigenic determinants that could be specifically recognized by HLA.A2-restricted CTL (Schendel et al, 1993; Bernhard et al, 1994).

In this study, HLA.A32 is the only restriction element that significantly correlates with clinical response to IL-2; however, differences in the expression of HLA.A3 between responders and non-responders have also been noted. The association between some MHC phenotypes and response to therapy strongly suggests that in vivo, IL-2 may enhance cellular-mediated immunity directed against a tumour antigen and that some MHC determinants may be more efficient than others for endogenous tumour antigen presentation.

In this series, although the number of patients is too small to conclude, the HLA phenotype did not influence the overall survival of the responding population. Furthermore, when considering the HLA.A32 determinant individually, among the nine patients who were A32-positive (11.4%), four did not respond to IL-2 therapy. For HLA.A3 allele, the proportion of non-responding patients (14/22) is even greater. Thus, this parameter alone is not sufficient to delineate the subgroup of patients that would benefit from IL-2 therapy. These data suggest that, in RCC, mechanisms other than the processing of tumour antigen can lead to immunosuppression and tumour progression. Although these mechanisms have not been completely elucidated, structural and functional alterations in lymphocytes infiltrating RCC tumours have been recently described. Other mechanisms, such as the production of immunosuppressive cytokines (Wang et al, 1995; Ménetrier-Caux et al submitted for publication), alterations in T-cell signal transduction (Finke et al, 1993) or an inefficient co-stimulation by accessory molecules (Bain et al, 1996), can also be put forward.

Prospective analyses of a larger series of patients are needed in order to validate these results.

### ACKNOWLEDGEMENT

This work was supported by a grant from the Rhône Committee of the French National League against Cancer.

### REFERENCES

Atzpodien J, Korfer A, Franks CR, Poliwoda H and Kirchner H (1990) Home therapy with recombinant IL-2 and recombinant interferon alpha 2B in advanced human malignancies. *Lancet* **335**: 1509–1512

Bain C, Merouche Y, Puisieux I, Duc A, Colombo MP and Favrot MC (1996) B7.1 gene transduction of human renal cell carcinoma cell lines restores the proliferative response and cytotoxic function of allogenic T cells. *Int J Cancer* **67**: 769–776

Bernhard H, Karbach J, Wölfel T, Busch P, Störkel S, Stöckle M, Wölfel C, Seliger B, Huber C, Meyer Sum Büschenfelde KH and Knuth A (1994) Cellular immune response to human renal-cell carcinomas: definition of a common antigen recognized by HLA-A2 restricted cytotoxic T-lymphocyte (CTL) clones. *Int J Cancer* **59**: 837–842

Blay JY, Négrier S, Combaret V, Attali S, Goillot E, Merrouche Y, Mercatello A, Ravault A, Tourani JM, Moskovtchenko, JF, Philip T and Favrot M (1992) Serum level of interleukin-6 as a prognosis factor in metastatic renal cell carcinoma. *Cancer Res* **52**: 3317–3322

Dellon AL, Rogentine Jr GN and Chretien PB (1975) Prolonged survival in broncogenic carcinoma associated with HL-A antigens W-19 and HL-A5: a preliminary report. *J Natl Cancer Inst* **54**: 1283–1286

Falk J and Osoba D (1971) HL-A antigens and survival in Hodgkin's disease. *Lancet* 1118–1121

Finke JH, Zea AH, Stanley J, Longo DL, Mizoguchi H, Tubbs RR, Wiltrout RH, O'Shea JJ, Kudoh S, Klein E, Bukowski RM and Ochoa AC (1993) Loss of T-cell receptor ζ chain and $p56^{lck}$ in T-cells infiltrating human renal cell carcinoma. *Cancer Res* **53**: 5613–5616

Kantor AF, McLaughlin JK, Blattner WA, Bach FH, Blot WJ, Schuman LM and Fraumeni JR JF (1983) HLA antigens in renal cell carcinoma. *Cancer Res* **43**: 2330–2333

Lilly F, Boyse EA and Old LJ (1964) Genetic basis of susceptibility to viral leukaemogenesis. *Lancet* 1207–1209

Marincola FM, Shamamian P, Rivoltini L, Salgaller M, Cormier J, Restifo NP, Simonis TB, Venzon D, White DE and Parkinson DR (1995) HLA associations in the antitumor response against malignant melanoma. *J Immunother* **18**: 242–252

Merrouche Y, Négrier S, Bain C, Combaret V, Mercatello A, Coronel B, Moskovtchenko JF, Tolstoshev P, Moen R, Philip T and Favrot MC (1995) Clinical application of retroviral gene transfer in oncology: Results of a French study with tumor-infiltrating lymphocytes transduced with the gene of resistance to neomycin. *J Clin Oncol* **13**: 410–418

Miller RG Jr (1981) *Simultaneous Statistical Inference*. Springer-Verlag New York

Mitchell MS, Harel W and Groshen S (1992) Association of HLA phenotype with response to active specific immunotherapy of melanoma. *J Clin Oncol* **10**: 1158–1164

Négrier S, Philip T, Stoter G, Fossa SD, Janssen S, Iacono A, Cleton FS, Israel L, Jasmin C, Rugarli C, Masse HVD, Thatcher N, Symann M, Bartsch HH, Bergmann L, Bijman JT, Palmer PA and Franks CR (1989) Interleukin-2 with or without LAK cells in metastatic renal carcinoma: a report of a European multicentric study. *Eur J Cancer Clin Oncol* **25**: S21–S28

Oliver RTD, Pillai A, Klouda PT and Lawler SD (1977) HLA linked resistance factors and survival in acute myelogenous leukemia. *Cancer* **39**: 2337–2341

Rosenberg SA, Lotze ML, Muul LM, Chang AE, Leitman S, Avis FP, Linehan WM, Robertson GM, Lee RE, Rubin JT, Seipp CA, Simpson C and White DE (1987) A progress report on the treatment of 157 patients with advanced cancer using lymphokine activated killer cells and interleukin-2 or high dose interleukin-2 alone. *N Engl J Med* **316**: 889–897

© Cancer Research Campaign 1997 

Rubin JT, Duquesnoy R, Simonis B, Adams S, Lee J and Lotze MT (1995) HLA-DQ1 is associated with clinical response and survival of patients with melanoma who are treated with interleukin-2. *Ther Immunol* **2**: 1–6

Schendel DJ, Gansbacher B, Oberneder R, Kriegmair M, Hofstetter A, Riethmüller G and Segurado OG (1993) Tumor-specific lysis of human renal cell carcinomas by tumor-infiltrating lymphocytes. *J Immunol* **151**: 4209–4220

Sheibenbogen C, Keilholz U, Mytilineos J, Suciu S, Manasterski M and Hunstein W (1994) HLA class I alleles and responsiveness of melanoma to immunotherapy with interferon-alpha (IFN-alpha) and interleukin-2 (IL-2). *Melanoma Res* **4**: 191–194

Wang Q, Redovan C, Tubbs R, Olencki T, Klein E, Kudoh S, Finke J and Bukowski RM (1995) Selective cytokine gene expression in renal cell carcinoma tumor cells and tumor-infiltrating lymphocytes. *Int J Cancer* **61**: 780–785

West WH, Taner KW, Yanelli JR, Marshall GD, Orr DW, Thurmann GB and Oldham RT (1987) Constant infusion recombinant IL-2 in adoptive immunotherapy of advanced cancer. *N Engl J Med* **316**: 898–905

© Cancer Research Campaign 1997